# Predatory *Bacteriovorax* Communities Ordered by Various Prey Species

**DOI:** 10.1371/journal.pone.0034174

**Published:** 2012-03-26

**Authors:** Huan Chen, Shanterial Young, Timkhite-Kulu Berhane, Henry N. Williams

**Affiliations:** School of the Environment, Florida A&M University, Tallahassee, Florida, United States of America; Uppsala University, Sweden

## Abstract

The role of predation in altering microbial communities has been studied for decades but few examples are known for bacterial predators. *Bacteriovorax* are halophilic prokaryotes that prey on susceptible Gram-negative bacteria. We recently reported novel observations on the differential selection of *Bacteriovorax* phylotypes by two different prey, *Vibrio parahaemolyticus* and *Vibrio vulnificus.* However, the conclusion is restricted by the limited number of prey tested. In this study, we have conducted two independent investigations involving eight species of prey bacteria while using *V. vulnificus* and *V. parahaemolytics* as reference strains. Water samples collected from Dry Bar, Apalachicola Bay were used to establish microcosms which were respectively spiked with prey strains *Vibrio cholerae*, *Escherichia coli* or *Pseudomonas putida* to examine the response of native *Bacteriovorax* to freshwater bacteria. Indigenous *Vibrio* sp., *Pseudoalteromonas* sp., *Photobacterium* sp. and a clinical strain of *V. vulnificus* were also tested for the impact of saltwater prey on the *Bacteriovorax* community. At 24 hour intervals, optical density of the microcosm samples and the abundance of *Bacteriovorax* were measured over five days. The predominant *Bacteriovorax* plaques were selected and analyzed by 16S rRNA gene amplification and sequencing. In addition, the impacts of prey on predator population and bacterial community composition were investigated using culture independent denaturing gradient gel electrophoresis. Strikingly, Cluster IV was found consistently as the predominant phylotype produced by the freshwater prey. For all saltwater prey, subgroups of *Bacteriovorax* phylotype IX were the major predators recovered. The results suggest that prey is an important factor along with temperature, salinity and other environmental parameters in shaping *Bacteriovorax* communities in aquatic systems.

## Introduction

Predation is an important service to the environment in maintaining population balances among organisms and food webs [1], [2]. The control of bacterial communities by predation has been known for several decades; however, the greatest progress in uncovering the types and roles of predators has occurred in the past 50 years with the discovery of *Bdellovibrio* and like organisms (*BALOs*) [3], millions of bacteriophage in aquatic systems [4], [5] and an improved understanding of the activities of protists [6]. Most investigations on bacterial predation have focused on the role of viruses and protists. The impact of BALOs in altering bacterial communities through predation is only beginning to be understood. Genera of these obligate predators share a unique life cycle consisting of two distinct phases, the predatory extracellular, attack phase in which the cells are highly motile to facilitate predation, and an intraperiplasmic growth phase in which they penetrate the cell wall and become lodged in the periplasmic space where they grow, multiply and finally free themselves by lysis of the prey cell [7]. Evidence strongly suggests they exert a potential sideways control, a mechanism by which bacterial predators prey on other prokaryotic or eukaryotic cells. In this way, they alter the structure, function, and dynamics of bacterial communities [8], [9].

BALOs attack Gram negative bacteria, however, not all are susceptible to the predators and among those that are, not all are preyed upon with equal efficiency [10], [11], [12]. We recently reported novel observations on the differential selection of *Bacteriovorax*, a saltwater genus of BALOs, by two different prey, *V. parahaemolyticus* and *V. vulnificus* [13]. When an environmental water sample was amended with high numbers of the respective prey, the progeny yield from *V. vulnificus* over a five day period was primarily restricted to two *Bacteriovorax* phylotypes, Clusters IX and X. Conversely, *V. parahaemolyticus* yielded multiple phylotype clusters, up to five in one case, which typically varied from day to day.

However, that study included only two bacteria prey, both from the same genus which limit the conclusions that can be drawn. To further explore this phenomenon we have conducted two independent investigations involving eight species of prey bacteria. *V. vulnificus* and *V. parahaemolyticus* were included as reference strains. Moreover, the impacts of the amended prey and subsequent increase in the predator population and bacterial community composition (BCC) were investigated using a culture independent approach. The results are described in this report.

## Materials and Methods

### Sample collection

Water samples were collected at site Dry Bar in Apalachicola Bay, Florida USA (N 29°40′13″; W85°05′39″) on three occasions designated as DB4, DB5 and DB6. On each occasion, bottom water was collected from both sides of a National Estuarine Research Reserve research vessel (25-foot, C-Hawk) using a sterile sampler at a depth of approximately 1.74 m. Environmental parameters were measured and recorded on site (Table 1). The water samples were stored on ice and transported to the laboratory at Florida A&M University for the setup of the microcosms within 6 h of collection. No specific permits were required for sampling in the above location.

**Table 1:** Measurements of environmental parameters of water samples collected to establish microcosm experiments.

Sampling ID	Temperature (°C)	Salinity (ppt)	pH	Dissolved Oxygen (mg L^−1^)	Purpose
DB4	17.4	14.1	8.3	7.6	Establishment of microcosms testing the impact of fresh water bacteria on *Bacteriovorax* community.
DB5	28.2	25.1	7.7	6.06	Isolation of prey bacteria indigenous to Dry Bar water.
DB6	24.5	24.4	8.2	5.79	Establishment of microcosms testing the impact of bacteria isolated from DB5 water on *Bacteriovorax* community

### Bacterial strains and culture conditions

Two prey species, *V. vulnificus* FLA042 (*Vv*) and *V. parahaemolyticus* strains P-5 (*Vp*) were included as reference strains in all microcosm experiments because their impact on shaping predator communities has been previously reported [13]. Other bacteria used in microcosm experiments included freshwater bacteria strains *Vibrio cholerae*, *Escherichia coli* or *Pseudomonas putida* which were selected from our laboratory culture collection and are known to be susceptible to *Bacteriovorax*. The saltwater bacteria used were indigenous species isolated from DB5 waters onto Luria-Bertani (LB) culture plates (Difco, Sparks, MD, USA). After obtaining pure cultures, DNA of the isolates was extracted by boiling and 16S rRNA fragments were PCR amplified, sequenced and blasted against NCBI database to obtain their phylogenetic identity. Subsequently, isolates of *Vibrio* sp., *Pseudoalteromonas* sp., and *Photobacterium sp.* were selected for the microcosm experiments for their known susceptibility to *Bacteriovorax*.

Prey suspensions used to amend the microcosms and for plating for *Bacteriovorax* recovery were prepared by adding 5 mL of sterile 70% artificial sea water (Instant Ocean Aquarium Systems, Inc., Mentor, OH, USA) (pH 8, Salinity 22 p.p.t.) to an overnight culture on LB plates. The bacterial colonies were suspended in the liquid for subsequent usage.

### Establishment of laboratory microcosms

For both experiments, water samples were mixed and filtered through a 0.8 µm filter to remove debris and larger organisms such as some protists for the establishment of the microcosms. Five hundred ml of the filtrate was dispensed into each of four 2 L Erlenmeyer flasks. For subsequent analysis by denaturing gradient gel electrophoresis (DGGE), another 500 ml of the filtrate was filtered through a 0.1 µm filter to capture the microbial populations, including *Bacteriovorax*, on the filters which were stored at −20°C.

To investigate the response of *Bacteriovorax* communities to the freshwater bacteria, microcosms established with DB4 water samples were amended with *V. cholerae*, *E. coli* or *P. putida*, respectively, in addition to the two reference strains, *Vv* and *Vp*.

To access the effect of salt water prey bacteria indigenous to Dry Bar water, *Vibrio sp.*, *Pseudoalteromonas sp.*, and *Photobacterium sp.* isolates were spiked into microcosms consisting of DB6 water. A clinical strain of *V. vulnificus* mo6 (*Vv*2) was also included in this experiment to test against reference strain *Vv* for strain-specific variations in selecting for *Bacteriovorax*.

Suspensions of the prey bacteria were spiked into the respective flasks described above to yield an optical density (OD) measurement of 0.7 at 600 nm except *Photobacterium sp.* of which OD was adjusted to 0.26. This corresponds to approximately 5×10^8^ cells ml^−1^ as predetermined by enumeration on LB agar plates. Control microcosms established to monitor prey abundance without interference from *Bacteriovorax* or other microorganisms consisted of equal volumes of prey as in the test microcosms in autoclave-sterilized environmental water. The microcosm flasks were shaken at room temperature and monitored at 24 h intervals through 120 h. Subsequent sample processing assays were adapted from Chen et al. [13]. Briefly, at each 24 h interval, OD measurements (at 600 nm) were taken of samples from the test and control microcosms. Aliquots were removed from test microcosms, serially diluted and plated using the double agar overlay technique [14] for isolation of predominant *Bacteriovorax* strains using the same prey as in the microcosm. Following incubation, plaque-forming units (PFU) were counted and recorded.

To identify the predominant population, *Bacteriovorax* plaques appearing on plates of the highest dilution were picked for 16S rRNA gene amplification using Bacteriovoracaceae specific primers (Bac-676F, Bac-1442R) [15]. This was followed by purification with the QIAquick PCR-Purification Kit (QIAGEN) and sequencing using Bac-676F primer at the DNA Sequencing Laboratory at Florida State University. DNA sequences and homology searches were analyzed with the Basic Local Alignment Search Tool (BLAST) server from the National Center of Biotechnology Information (www.ncbi.nlm.nih.gov). Phylogenetic clusters of *Bacteriovorax* were assigned based on 96.5% or higher 16S rRNA gene sequence similarities with the strains described in previous reports [9], [16], [17]. Complementary to this cultural dependent technique, denaturing gradient gel electrophoresis (DGGE) was also used to monitor shifts in the *Bacteriovorax* and bacterial communities in the microcosms using universal bacterial primer GM5F-GC and 907R. DGGE bands representing the most prominent operational taxonomic units (OTUs) were excised and the DNA eluted and re-amplified using primers without the GC clamp and sequenced. These sequences were checked for chimeras using the Bellerophon Chimera Check (version 3) [18] and have been submitted to the GenBank databases under accession numbers (JQ612074-JQ612128). The sequences were taxonomically characterized by Basic Local Alignment Search Tool (BLAST).

### Data analyses

The abundance of predator and prey (log transformed) were analyzed by analysis of variance (ANOVA) to detect significant differences among the numbers of bacteria in the various microcosm treatments. When ANOVA tests were passed, the Holm-Sidak test was performed. The T-test was used to compare two groups of treatments when normality and equal variance tests were passed. All statistical analyses were performed using the Sigmastat, version 3.5, software package.

For DGGE analysis, Quantity-one software (version 4.0, Bio-Rad, USA) was used to determine the presence, intensity and relative position of each band. Each DGGE band was assumed to represent an OTU or phylotype.

## Results

### Reduction in prey abundance by *Bacteriovorax* predation

**Figure 1:**
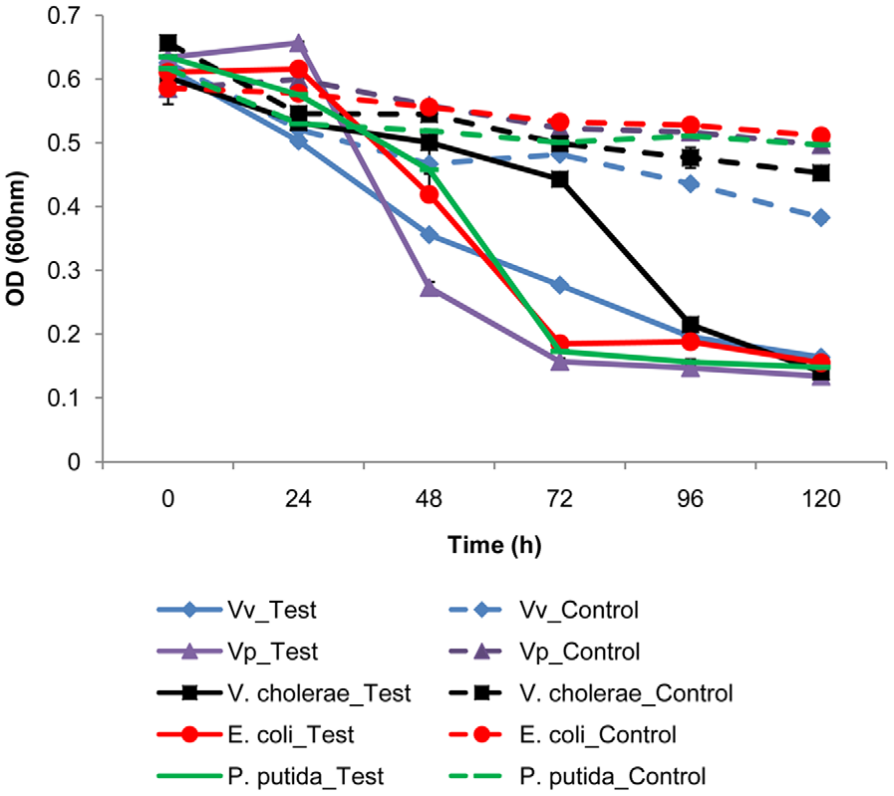
Kinetics of the lysis of freshwater prey species and reference strains (*Vv*,*Vp*) by *Bacteriovorax*. Both test (with *predators*) and control (without *predators*) microcosms were established in DB4 water and measurements of cell density in both were taken by OD. Bars indicate standard errors of the mean of three replicates; in some cases bars are too small to be visible.

**Figure 2:**
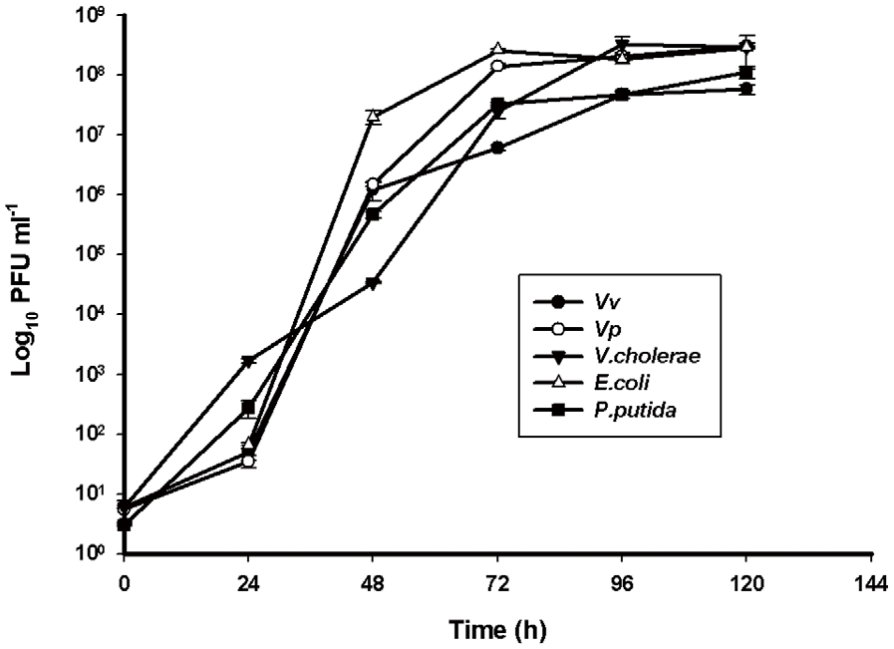
Numbers of *Bacteriovorax* from microcosms amended with three freshwater bacteria and the reference strains (*Vv*,*Vp*) respectively. Microcosms were established in DB4 waters. Samples were taken at various time intervals. Bars indicate standard errors of the mean (N = 3).

**Figure 3:**
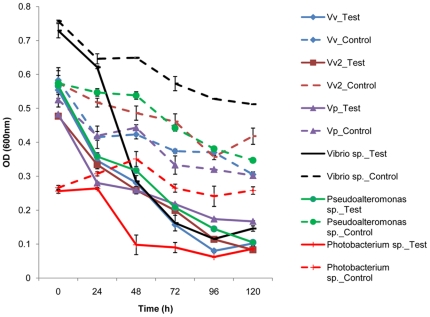
Kinetics of the lysis of indigenous saltwater prey by *Bacteriovorax* over time. Both test (with *predators*) and control (without *predators*) microcosms were established in DB6 water and cell density was measured by OD. Bars indicate standard errors of the mean (N = 3).

**Figure 4:**
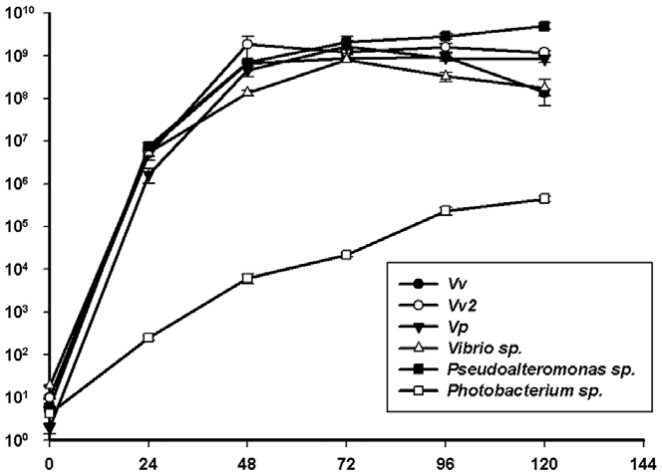
Numbers of *Bacteriovorax* from the microcosms established with native bacteria and reference strains (*Vv*,*Vp*), respectively. Microcosms were established in DB6 water. Bars indicate standard errors of the mean (N = 3).

In the two independent experiments with laboratory microcosms, the predator and prey responses exhibited similar patterns (Fig. 1, 2, 3, 4). In all cases, the inoculated prey bacteria decreased significantly (ANOVA, p<0.01) with a simultaneous increase in *Bacteriovorax* numbers, indicating the predation of the spiked prey by the predator. These results are consistent with that of our previous report [13]. The greatest decreases in OD measurements were generally observed after 24 to 48 h except for the microcosms spiked with *V. cholerae*, which occurred after 72 h indicating a delayed response of *Bacteriovorax* to this prey (Fig. 1). As expected, the initial concentrations of *Bacteriovorax* in the water samples before adding the test prey were very low, ranging from below detectable levels up to 10 PFU ml^−1^. However, the *Bacteriovorax* population grew rapidly on the spiked bacteria. In microcosms with freshwater prey, *Bacteriovorax* numbers peaked around 10^8^ PFU ml^−1^ at 72 h and remained relative constant until 120 h (Fig. 2). This is a typical response of the predators to high concentrations of prey bacteria and shows their ability to rapidly increase in number going from a rare dormant species to a dominant active population to control and reduce the prey population. The *Bacteriovorax* numbers in microcosms with saltwater prey also showed similar trends, albeit at a higher growth efficiency as revealed by a sharp increase within the first 24 h that peaked after 48 h of incubation. Notably, *Bacteriovorax* grew at a slower rate on *Photobacterium sp.*, gradually reaching 10^6^ PFUs ml^−1^ after 120 h of inoculation (Fig. 4).

### Analysis of predator communities amended with freshwater prey

**Figure 5:**
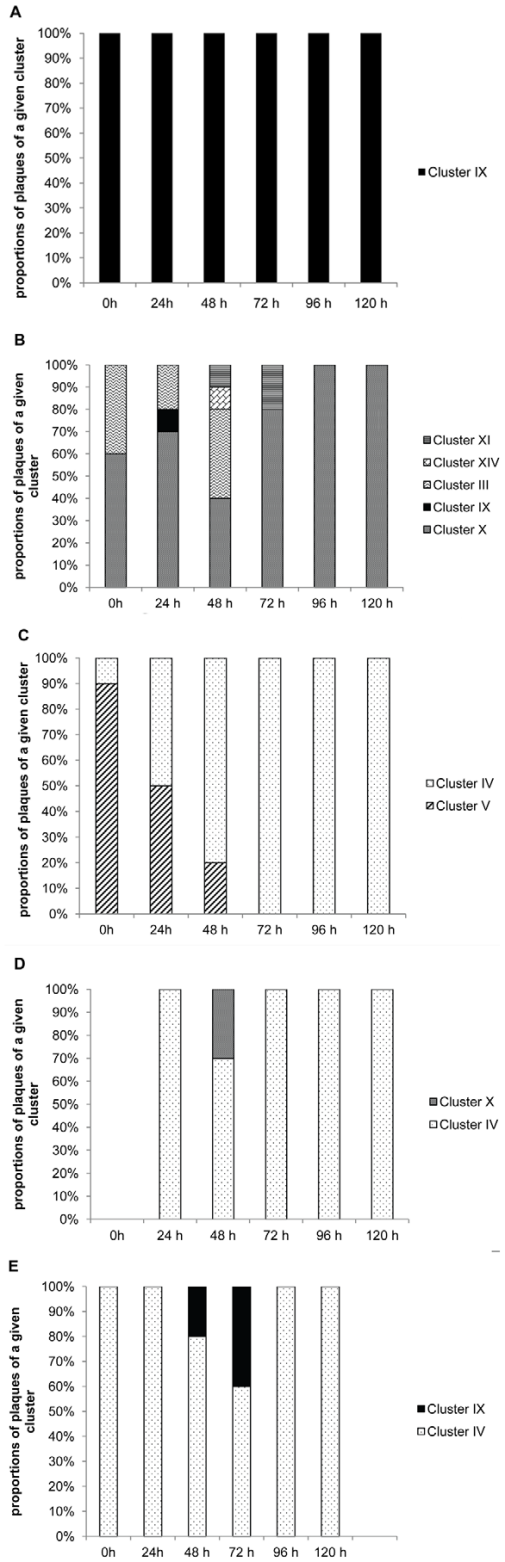
Predominant *Bacteriovorax* OTUs recovered from the microcosms established with freshwater prey and reference strains. Microcosms were amended with *Vv* (A), *Vp* (B), *V. cholera* (**C**), *E. coli* (D) and *P. putida* (E). Clusters based on 96.5% 16S rRNA gene sequence similarity are numbered consistently with previous reports [9], [16], [17].

*Bacteriovorax* phylogenetic Cluster IV was the predominant predator population emerging from the three freshwater prey (Fig. 5). Strikingly, Cluster IV was not recovered from the microcosms of the two reference prey, *Vv* nor *Vp*, (Fig. 5 a, b). This is consistent with our previous report that *Vv* typically and consistently selected for *Bacteriovorax* Cluster IX throughout the experiment *Vv* whereas with *Vp* the predominant predator phylotype population was less stable changing on a nearly daily basis. The number and diversity of phylotypes were also higher with several phylotypes being represented among those that were dominant. *Bacteriovorax* population was observed in *Vp* microcosms. Although the dominant *Bacteriovorax* recovered from all three fresh-water prey belonged to Cluster IV, the patterns were still slightly different. *Bacteriovorax* Cluster V appeared in the *V. cholerae* microcosm during the first 48 h and was gradually taken over by Cluster IV (Fig. 5 c). *Bacteriovorax* phylotype Cluster X appeared in high abundance at 48 h in *E. coli* microcosm (Fig. 5 d) and Cluster IX was found in *P. putida* microcosm at 48 h and 72 h (Fig. 5 e).

### DGGE analysis of microcosms established with freshwater bacteria in DB4 water

**Figure 6:**
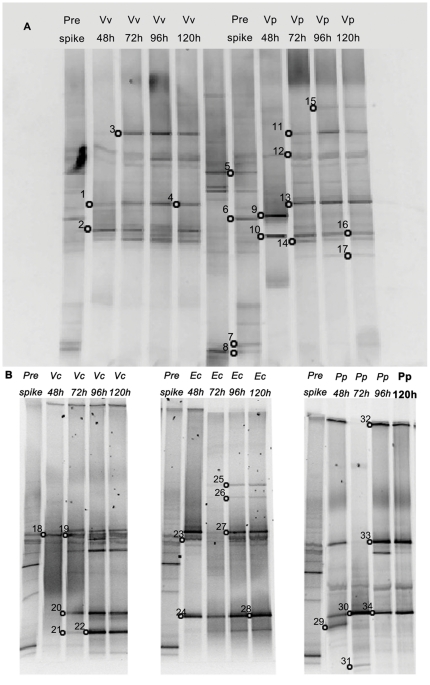
Analyses of DGGE banding patterns (PCR-amplified 16S rRNA gene fragments) in microcosms amended with freshwater species and the reference strains. Microcosms were established in DB4 water and samples were taken at various time points. (A) Microcosms amended with *Vv* and *Vp* as reference prey. (B) Microcosms established with *V. cholera* (Vc), *E. coli* (Ec) and *P. putida* (Pp). Lanes labeled pre-spike, 48 h, 72 h, 96 h and 120 h indicate the time points at which the samples were removed from the microcosm. Open circles indicate the excised and sequenced bands.

A total of 32 prominent bands in the DGGE gel images were excised and sequenced (position shown in Fig. 6). The major predators responsible for *Vv* reduction are clustered to phylotype IX. For *Vp* microcosm, a prominent band (band 9) related to Cluster III is shown at 48 h, however, at later time points Cluster X was predominant (band 13). Cluster XI was also found in *Vp* microcosms (band 17). Bands related to Cluster IV were detected in *V. cholerae*, *E. coli* and *P. putida* microcosms (band 20, 24, 28, 30 and 34). Bands related to *Bacteriovorax* Cluster V were observed only in *V. cholerae* microcosms (band 21 and 22). Cluster IX was only detected in the *P. putida* microcosm at 72 h (Table 2). The results reveal that the predator phylotypes profiled by DGGE are consistent with that of the culture dependent method described above.

**Table 2:** Identification of bacteria (based on 16S rDNA sequence similarity to the nearest neighbor from NCBI database) in samples retrieved from microcosms established with freshwater bacteria in DB4 water (See Fig. 6 for position of the bands).

DGGE Band No.	Taxonomic classification	Most closely related sequence	Identity (%)	Accession no. of related sequence
1 (4)	*Bacteriovorax* Cluster IX	*Bacteriovorax* sp. OC51	99	DQ631726.1
2	*Vibrio vulnificus*	*Vibrio vulnificus* CMCP6	99	AE016796.2
3	Bacteroidetes	Uncultured Bacteroidetes bacterium clone C319a-R8C-D4	94	AY678514.1
5	Unclassified Bacteria	Uncultured marine bacterium clone IMS3D2-37	99	JN233702.1
6	Beta proteobacterium	Beta proteobacterium BAL58	98	AY317112.1
7	Unclassified_Micrococcineae	Uncultured Actinomycetales bacterium clone SHWH_night1_16S_564	98	FJ744815.1
8	Actinomycetales	Uncultured marine bacterium clone 29-B40	96	GU576917.1
9	*Bacteriovorax* Cluster III	*Bacteriovorax* marinus SJ genome	99	FQ312005.1
10 (16)	Vibrio parahaemolyticus	Vibrio parahaemolyticus isolate VP332	99	JF779841.1
11	Bacteroidetes	Uncultured Bacteroidetes bacterium clone C319a-R8C-D4	94	AY678514.1
12	Tenacibaculum mesophilum	Tenacibaculum mesophilum strain HNS042	100	JN128276.1
13	*Bacteriovorax* Cluster X	*Bacteriovorax* sp. BB1	98	DQ631713.1
14	Thalassospira sp.	Thalassospira xianheensis strain PM01	99	HM587995.1
15	Flavobacterium sp.	Flavobacterium sp. FCS-5	95	JF830803.1
17	*Bacteriovorax* Cluster XI	*Bacteriovorax* sp. MIA4	100	DQ631697.1
18 (19)	Vibrio cholerae	Vibrio cholerae strain SX-1	99	JN555611.1
20	*Bacteriovorax* Cluster IV	*Bacteriovorax* sp. OC91	100	DQ631737.1
21 (22)	*Bacteriovorax* Cluster V	*Bacteriovorax* sp. JS81	99	DQ631738.1
23	*Escherichia coli*	Escherichia coli strain NCTC 50271	100	JN654456.1
24 (28)	*Bacteriovorax* Cluster IV	*Bacteriovorax* sp. OC91	100	DQ631737.1
25	Flexibacter sp.	Uncultured Flexibacter sp.	95	FN668192.2
26	Coccinistipes sp.	Coccinistipes vermicola strain IMCC1411	97	EF108212.1
27	Bacteroidetes	Uncultured Bacteroidetes bacterium	94	FR670479.1
29	Pseudomonas putida	Pseudomonas putida strain LCB43	98	JN650580.1
30	*Bacteriovorax* ClusterIV	*Bacteriovorax* sp. DF2	99	EF092437.2
31	*Bacteriovorax* Cluster IX	*Bacteriovorax* sp. OC51	99	DQ631726.1
32	Flavobacterium sp.	Flavobacterium sp. FCS-5	97	JF830803.1

Other bacterial species were also identified in the PCR-DGGE gel bands. *Bacterioidetes* were observed to thrive in all the DB4 microcosms after prey inoculation. The *Vv* and *Vp* amended microcosms enhanced the growth of *Thalassospira sp.* (Fig. 6. a), whereas, the *E. coli* uniquely facilitated the growth of *Flexibacter sp.* and *Coccinistipes sp.* (Fig. 6 b).

### Analysis of predator community arising from saltwater indigenous prey bacteria

**Figure 7:**
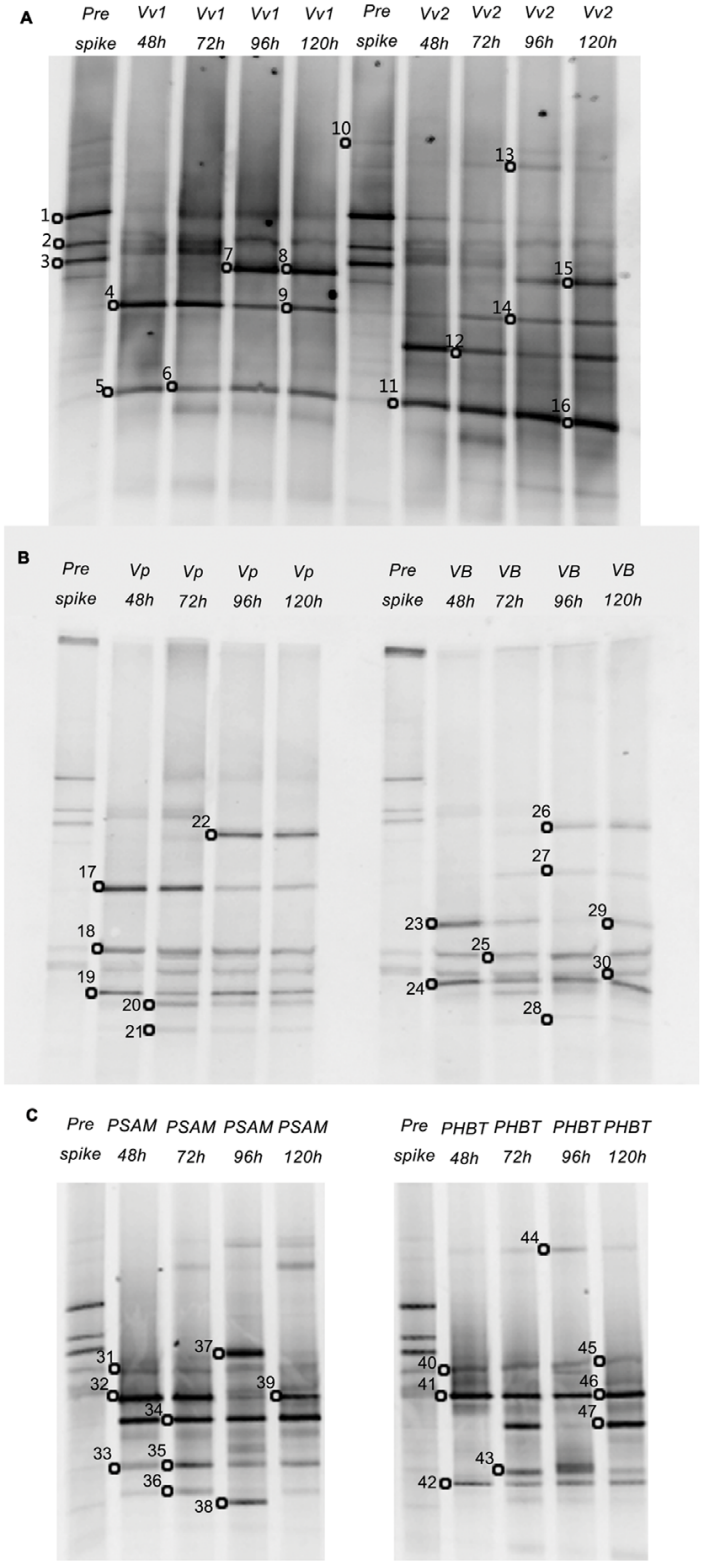
Analyses of DGGE banding patterns (PCR-amplified 16S rRNA gene fragments) in microcosms established with indigenous prey species and reference strains. Microcosms were established in DB6 water and samples were taken at various time points. (A) Microcosms spike with *Vv* and *Vv*2. (B) Microcosms spiked with *Vp* and *Vibrio sp.* (VB). (C) Microcosms inoculated with *Pseudoalteromonas sp.* (PSAM) and *Photobacterium sp.* (PHBT). Open circles indicate the excised and sequenced bands.

Strikingly, *Bacteriovorax* Cluster IX was the predominant predator recovered by the culture method in microcosms established with the saltwater native prey bacteria. The DGGE banding patterns of the microbial populations in these same microcosms are shown in Fig. 7. DNA sequences obtained from a total of 47 bands on the DGGE gels were used to determine phylogenetic analysis. The nearest neighbor and phylogenetic groups of each sequence are listed in Table 3. Consistent with the results of plaque analyses, bands related to *Bacteriovorax* phylotype IX were present in all microcosms. In addition, *Bacteriovorax* Cluster XII was found in *Pseudoalteromonas sp.* microcosm at 72 h and Cluster XIII was found in *Photobacterium sp.* microcosm after 48 h. Interestingly, another clade of *Bacteriovorax* phylotype which shared a similarity index of 89% with *Peredibacter* sp., a freshwater BALO sp., was also revealed by DGGE analysis (bands 15, 22, 26 and 37). This clade was identified in all microcosms after 72 h except for the one amended with *Photobacterium sp*.

**Table 3:** Identification of bacteria (based on 16S rDNA sequence similarity to the nearest neighbor from NCBI database) in samples retrieved from microcosms established with indigenous saltwater prey species in DB6 water (See Fig. 7 for position of the bands).

DGGE Band No.	Taxonomic classification	Most closely related sequence	Identity (%)	Accession no. of related sequence
1	Unclassified Bacteria	Uncultured marine bacterium clone IMS3D2-37	98	JN233702.1
2	Ruegeria sp.	Ruegeria sp. 1444	97	JN679843.1
3	Unclassified Bacteria	Uncultured bacterium clone 2CE2-5m-92	98	GU062170.1
4(9)	*Bacteriovorax* ClusterIX	*Bacteriovorax* sp. VAB51	100	DQ631747.1
5(6)	Vibrio vulnificus	Vibrio vulnificus strain VV11 16S ribosomal RNA gene	99	HQ341792.1
7(8)	Peredibacter	Uncultured bacterium clone N701B_78	99	GU941078.1
10	Unclassified Flavobacteriaceae	Uncultured bacterium clone D05JOA	95	JF692410.1
11(16)	Vibrio vulnificus	Vibrio vulnificus strain VV11	100	HQ341792.1
12	Ruegeria sp.	Ruegeria sp. 1444	99	JN679843.1
13	Unclassified Bacteria	Uncultured marine bacterium clone IMS3D2-37	98	JN233702.1
14	*Bacteriovorax* ClusterIX	*Bacteriovorax* sp. VAB51	99	DQ631747.1
15	Peredibacter	Uncultured bacterium clone N701B_78	99	GU941078.1
17	*Bacteriovorax* Cluster IX	*Bacteriovorax* sp. VAB51	99	DQ631747.1
18	*Bacteriovorax* ClusterIX	*Bacteriovorax* sp. VAB51	96	DQ631747.1
19	Vibrio parahaemolyticus	Vibrio parahaemolyticus isolate *Vp*011	99	EU155526.1
20(24)	Thalassospira sp.	Thalassospira sp. DG1243	95	DQ486488.1
21	Thalassospira sp.	Rhodospirillaceae bacterium EZ54	99	EU704115.1
22	Peredibacter sp.	Uncultured bacterium clone N701B_78	99	GU941078.1
23(29)	Ruegeria sp.	Ruegeria sp. 1444	99	JN679843.1
24	Vibrio sp.	Vibrio communis strain J821	99	JF836185.1
25	*Bacteriovorax* ClusterIX_96%	*Bacteriovorax* sp. VAB51	96	DQ631747.1
26	Peredibacter	Uncultured bacterium clone N701B_78	99	GU941078.1
27	*Bacteriovorax* Cluster IX	*Bacteriovorax* sp. VAB51	99	DQ631747.1
28	Thalassospira sp.	Thalassospira profundimaris strain mj01-PW1-OH20	98	HQ425693.2
30	Ruegeria sp.	Ruegeria sp. 1444	97	JN679843.1
31	Pseudoalteromonas sp.	Pseudoalteromonas sp. IAJ17	100	JN391176.1
32(39)	*Bacteriovorax* Cluster IX	*Bacteriovorax* sp. VAB51	99	DQ631747.1
33(35)	Thalassospira sp.	Rhodospirillaceae bacterium EZ54	99	EU704115.1
34	Ruegeria sp.	Ruegeria sp. 1444	99	JN679843.1
36	Thalassospira sp.	Thalassospira profundimaris strain mj01-PW1-OH20	99	HQ425693.2
37	Peredibacter	Uncultured bacterium clone N701B_78	98	GU941078.1
38	*Bacteriovorax* Cluster XII	*Bacteriovorax* sp. HAWAII2	99	DQ631769.1
40 (45)	*Bacteriovorax* ClusterIX	*Bacteriovorax* sp. VAB51	98	DQ631747.1
41 (46)	*Bacteriovorax* Cluster IX	*Bacteriovorax* sp. VAB51	99	DQ631747.1
42	Photobacterium sp.	Photobacterium sp. UST991130-005	98	AF465393.1
43	*Bacteriovorax* Cluster XIII	*Bacteriovorax* sp. MER21	98	DQ631740.1
44	Roseivirga sp.	Roseivirga spongicola strain UST030701-084	98	NR_043531.1
47	Ruegeria sp.	Ruegeria sp. 1444	100	JN679843.1

Bacteria other than the prey and predators in the microcosms were detected by DGGE. *Ruegeria* sp. was detected from all microcosms except for the *Vv* microcosm. *Thalassospira* sp. was detected in *Vp*, *Vibrio sp.* and *Pseudoalteromonas sp.* microcosms but not in the *Vv*, *Vv2* and *Photobacterium sp.* microcosms. Notably, sequences affiliated with the phylum Bacterioidetes were abundant in microcosms established with freshwater bacteria but were not detected in this experiment with indigenous prey. A sequence closely related to *Roseivirga* sp. 16S rDNA was found in the *Photobacterium sp.* microcosm (band 44).

## Discussion

Previously, we reported the first direct evidence of the influence of prey bacteria on selection of predatory *Bacteriovorax* phylotypes. In that study we observed that two prey species of the same genus, *Vv* and *Vp* [13] typically yielded different *Bacteriovorax* phylotypes, although both were added to the same environmental sample and thus available to the same native predator population. This result was consistent across water bodies of varying salinities, including ocean, estuarine and the Gulf of Mexico, water temperatures and seasons.

In the current study, we conducted a more comprehensive investigation which included more bacteria of different genera with representatives from both freshwater and saltwater environments. *Vv* and *Vp* were also included as reference strains since we had prior knowledge of their impact from our previous study. The results confirmed our previous findings that the bacterial prey of *Bacteriovorax* influences the strains of predators that multiply within them and are released into the environment when the prey cell is lysed. Another surprising observation made was a distinct difference between the *Bacteriovorax* population produced by the freshwater and saltwater test prey bacteria. The three freshwater prey bacteria consistently yielded *Bacteriovorax* phylotype Cluster IV as the predominant predator. In contrast, Cluster IV was not found in the microcosms amended with the reference halophilic strains, *Vv* and *Vp* (Fig. 5). This is interesting since Cluster IV was consistently isolated using *Vp* as prey from low salt waters of the Chesapeake Bay. However, since Cluster IV is typically found in low salt aquatic environments it may encounter both fresh and salt water prey bacteria and has become adapted to preying on both, but apparently, in this case, more efficiently on the freshwater strains.

We also observed that *Bacteriovorax* Cluster V was only detected in the *V. cholerae* microcosm. Cluster V, like cluster IV, is a distinct estuarine strain which has been recovered only from low salinity areas of Chesapeake Bay and other estuarine systems , but not from marine or high salt ecosystems [19], [20]. Perhaps Cluster V has a preference for freshwater prey such as *V. cholerae* and may thrive best in those regions where the preferred prey is present.

Contrary to the *Bacteriovorax* Clusters IV and V produced from the freshwater prey, the predominant predator phylotype recovered from the halophilic prey was Cluster IX. Isolates of Cluster IX have been reported to be ubiquitous and the most abundant *Bacteriovorax* group cultured from marine environments [19]. Wen et al, [21] attributed their ability to adapt to saltwater environments to the heterogeneity of their 16S rRNA operons, as only Cluster IX displayed multiple visible V3 bands in DGGE gels. This was also observed in our study which, in one case, showed that two DGGE bands from the same sample were related to Cluster IX with 16S rRNA percentage similarities of 99% and 96% (Fig. 7b, bands 17and 18). This unique characteristic of Cluster IX may explain their superiority in controlling halophilic prey species.

Previous reports have revealed that freshly isolated autochthonous bacteria appeared to be more lucrative prey for BALO isolates from the same habitat than laboratory maintained prey strains [22], [23], [24], [25] from other sources. Our results support this conclusion as greater prey reduction was observed in the microcosms spiked with the native *Vibrio sp.* than in the microcosms amended with the three laboratory *Vibrio* strains, *Vv*, *Vv*2 and *Vp* (Fig. 3).

It warrants noting that in microcosms established with indigenous prey, another clade of BALO phylotype which clustered within *Peredibacter*, a freshwater genus of BALOs, was also revealed by DGGE analysis (Table 3). Chauhan et al., [25] also discovered this clade in water samples from Apalachicola Bay which were bio-stimulated with yeast extract. The fact that the bands only were detected in microcosms after 72 h implies that the growth of this group may be slower in salt water environments. Their later growth and appearance may have been stimulated by an increase of available organic matter in the microcosms resulting from the massive lysis of prey by other *Bacteriovorax* phylotypes. If this clade is indeed a member of freshwater BALO, it also suggests it is a more versatile predator which has higher salt tolerance than the freshwater strains which are typically restricted by salt concentrations as low as 0.5%.

Our results demonstrate that not only are environmental physicochemical pressures among the factors which determine BALO phylotypes recovered from nature, but also the prey community upon which they feed. Moreover, not all phylotypes can be detected by a single, particular prey. BALOs which have either a different prey preference than the bacterium used in isolation or are uncultivable will remain unrepresented in biodiversity studies.

Both culture dependent and independent methods were applied in this study to detect BALO phylotypes and were found to result in detection of more phylotypes than either method alone. Typically the predator communities revealed by the two methods were largely consistent although slight individual variations were observed. For example, a more diverse *Bacteriovorax* community was depicted by culture methods than by DGGE in DB4 microcosm spiked with *Vp* (Fig. 5 b), whereas, in the DB6 water microcosm Clusters XIII and XII were detected only by the DGGE method (Table 3 bands 38, 43). One advantage of using DGGE with a universal primer is that it is not limited to detecting known *Bacteriovorax* taxa and therefore can identify novel BALO isolates. Using DGGE, a BALO clade related to *Peredibacter* was detected in DB6 water that could not be amplified with the Bac-specific primer. Overall, both methods when combined yielded a more detailed structure of the predator community, but it should be noted that they are only able to detect predominant members represented in the samples.

In the study, we also used DGGE profiling to evaluate the temporal variations in the total bacterial community structure following prey inoculation into the microcosms. The bacteria that became abundant after prey inoculation were *Bacterioidetes*, *Ruegeria* sp. and *Thalassospira* sp. *Bacteriodetes* are common microbes in coastal waters especially during algal blooms and are known for their ability to rapidly degrade complex dissolved organic matter (DOM) [26], [27]. It is also not surprising to find *Ruegeria* sp. and *Thalassospira* sp. predominant in the microcosm. Both are groups of alpha-Proteobacteria which is also a major component of BCC in marine environments dominating particularly in low nutrient conditions [28]. It should also be noted that both genera are gram-negative bacteria. Since *Bacteriovorax* preferentially prey on certain gram negative bacteria, it may provide the opportunity for *Bacteriovorax* resistant bacteria to thrive. Interestingly, even prey of the same species (*Vv* and *Vv*2) caused BCC to vary (Fig. 7 a). For instance, bands related to *Ruegeria* sp. were identified in the *Vv*2 microcosm but not in the *Vv* microcosm (Table 3, band 13). This suggests that predator-prey interaction has a complex role in regulating BCC and the impact is strain specific.

These results should be interpreted with some caution as the numbers of selected prey introduced into the natural water microcosms were several fold higher than typically detected in environmental waters. This may have influenced the population dynamics of the other bacteria in a way that may not be observed when the prey numbers are within their natural range. It was necessary to use such large numbers of prey in this study to amplify and detect the response of the *Bacteriovorax* community. Although the typical numbers of bacteria and prey in the water column may be below the threshold for highly active *Bacteriovorax* predation, there are instances in which spikes in bacterial populations occur, for example in the environment niches, sediments and biofilm, and events such as phytoplankton blooms, sewage input and decomposition of dead plants or animals. Under these circumstances, we believe the *Bacteriovorax* and other BALOs respond as we have observed in the microcosms in this study, by aggressively attacking and killing its prey until the prey population is substantially reduced. Although BALO numbers are typically very low, and sometimes undetectable, in environmental waters, the results of this and other studies [13] have shown the predators are able to rapidly increase by seven or eight logs in response to high numbers of prey bacteria.

The results from this study of eight bacteria representing different genera and species and our previous report [13] confirm that prey bacteria play an important role in determining the community composition and structure of *Bacteriovorax* communities in salt water systems. This particular function of prey bacteria was not known previously and unveils another aspect of the mystique and complexity of the predator-prey relationship of the BALOs and bacteria that serve as their prey. Further, the results revealed distinct differences in the *Bacteriovorax* phylotype communities derived from growth of the predators on freshwater and saltwater prey bacteria. Also, this study confirmed that in terms of methods for investigating *Bacteriovorax* native communities, the most accurate approaches include the use of both culture and non culture molecular techniques.

Since our studies were performed using microcosms made from natural water and the native bacterial population, it provided the opportunity to observe changes in the total bacterial population and especially those that may have been influenced by the amended prey and emerging BALO population. The collective results represent a major advance in the understanding of *Bacteriovorax* predator-prey interactions. Future studies are necessary to gain greater understanding of the mechanisms which governs the observed selective predation which leads to production of diverse *Bacteriovorax* population.
